# Two cases of HIVAN in young AIDS patients

**DOI:** 10.1186/1471-2334-14-S4-P38

**Published:** 2014-05-29

**Authors:** Rodica Costa, Mihai Gafencu, Raluca Isac, Valeria Bică, Gabriela Doroş

**Affiliations:** 1 HIV Department, Clinical Emergency Children’s Hospital “Dr. L. Turcanu”, Timişoara, Romania; 2 Department of Pediatrics, Dr. Victor Babeş University of Medicine and Pharmacy, Timişoara, Romania; 3 Laboratory – Infectious Disease Hospital Timişoara, Romania

## 

HIV associated nephropathy (HIVAN) is a quite frequent pathology among HIV infected patients with a high incidence in black people. Among the 800 patients from Western Romania, infected with HIV type 1, mostly subtype F, none were diagnosed based on renal biopsy with HIVAN until 2010, albeit several renal abnormalities have been described among HIV patients based on a complex etiology.

We present two cases, both Caucasian, a 25 years old female and a 26 years old male, HIV infected in the early 1990`s with horizontal transmission. First case was diagnosed with HIV infection as late-presenter and staged C3 at the age of 10 when she was admitted in coma secondary to toxoplasmic encephalitis. The first manifestations of nephropathy were detected 6 years later with decreased creatinine clearance. The second case was HIV diagnosed at the age of 19, in 2007, during hospital admission for acute glomerulonephritis with secondary renal impairment, as a late-presenter staged also C3. The patient presented hepatitis B co-infection as well as chronic CMV infection. Renal biopsy was performed on both patients and revealed aspects of focal and segmental glomerulosclerosis, applicable for HIVAN. Both patients started HAART immediately after diagnosis, none of the medications used had been showed to induce renal impairment and both of them had creatinine clearance adjusted dosing of antiretroviral (ARV) treatment, but in spite of similar ARV and supportive treatment, the two cases had different outcome. One had a very slow rate of decrease in renal function while the second one (similar to literature data) had a rapid evolution towards chronic kidney disease, within 3 years dialysis had to be initiated.

Compliance to antiretroviral treatment improves survival rate globally (with presumable late onset for chronic kidney disease). Renal biopsy remains the standard in order to diagnose HIVAN. As far as patients are aging with AIDS, renal manifestations may become more frequent and a comprehensive oversight is needed.

